# Crystal structure of duck egg lysozyme isoform II (DEL-II)

**DOI:** 10.1186/s12900-018-0090-7

**Published:** 2018-08-22

**Authors:** David B. Langley, Daniel Christ

**Affiliations:** 1 Immunology Division, Garvan Institute of Medical Research, 384 Victoria Road, Darlinghurst, Sydney, NSW 2010 Australia; 2 The University of New South Wales, Faculty of Medicine, St Vincent’s Clinical School, Darlinghurst, Sydney, NSW 2010 Australia

**Keywords:** Duck egg lysozyme, Lysozyme, Structure, DEL-II

## Abstract

**Background:**

Lysozyme purified from duck eggs (DEL) has long been used as a model antigen as a counterpoint to the enzyme purified from hen eggs (HEL). However, unlike the single C-type variant found in hen eggs, duck eggs contain multiple isoforms: I, II and III. We recently reported the structures of isoforms I and III from Pekin duck (*Anas platyrhynchos*) and unequivocally determined the sequences of all three isoforms by mass spectrometry. Here we present the crystal structure of isoform II (DEL-II).

**Results:**

Lysozyme isoform II was purified from isoforms I and III using ion-exchange and gel-filtration chromatography, then crystallized. X-ray diffraction data were collected to 1.15 Å resolution and the structure of DEL-II was solved by molecular replacement using the structure of DEL-I as the search model. It contains two molecules in the crystallographic asymmetric unit: both molecules display a canonical C-type lysozyme fold and electron density consistent with the expected sequence. The most significant difference between the two molecules concerns different conformations of a surface loop containing one of the expected amino acid differences between the isoforms.

**Conclusions:**

The structure of DEL-II supports the primary sequence as elucidated by a combination of amino acid sequencing, DNA sequencing and mass spectrometry, with strong electron density confirming it to be an S37G G71R variant of DEL I, and differing from hen egg lysozyme at a total of 21 amino acid positions.

**Electronic supplementary material:**

The online version of this article (10.1186/s12900-018-0090-7) contains supplementary material, which is available to authorized users.

## Background

While lysozymes purified from the eggs of chickens and ducks were both extensively studied throughout the 1960s, only the crystal structure of hen egg lysozyme (HEL) was reported [1]. This was no doubt aided by the fact that samples from duck eggs contained multiple isoforms (likely three), with amino acid analysis suggesting that isoforms were distinguished largely on the basis of containing different numbers of arginine residues [2, 3]. Although no structure of duck egg lysozyme (DEL) subsequently emerged, over the ensuing decades duck lysozyme was nevertheless intensely utilized in immunology. In particular, DEL was used as a model antigen juxtaposed against HEL, from which it differed at approximately 20 of the 129 amino acid positions (‘approximately’, as amino acid sequences derived via Edman Degradation for different isoforms and from different strains by different groups disagreed at a handful of positions (see Langley et al.*,* 2017 for a review)). Duck lysozyme, often strain and isoform unspecified, has been used to advance our understanding of many immunological phenomena including: antibody-antigen interactions [4, 5]; immune tolerance [6]; complement activation [7]; germinal center B-cell affinity maturation [8, 9]; T-follicular helper cell differentiation [10]; and, most recently, to dissect self/foreign discrimination during emergence from B-cell anergy [11].

The DNA sequence for one of the three Pekin duck isoforms (DEL-II) was finally published in 2013 [12], differing at two positions (both asparagine/aspartic acid discrepancies) with the sequence determined using Edman Degradation [13]. We recently used a combination of mass spectrometry and X-ray crystallography to unequivocally delineate sequences of all three Pekin DEL isoforms (DELs -I, -II and -III). However, we were only able to determine high-resolution X-ray structures of two isoforms (DEL-I and DEL-III) [14]. Here we report the high-resolution structure of the remaining isoform, DEL-II.

## Results

**Fig. 1:**
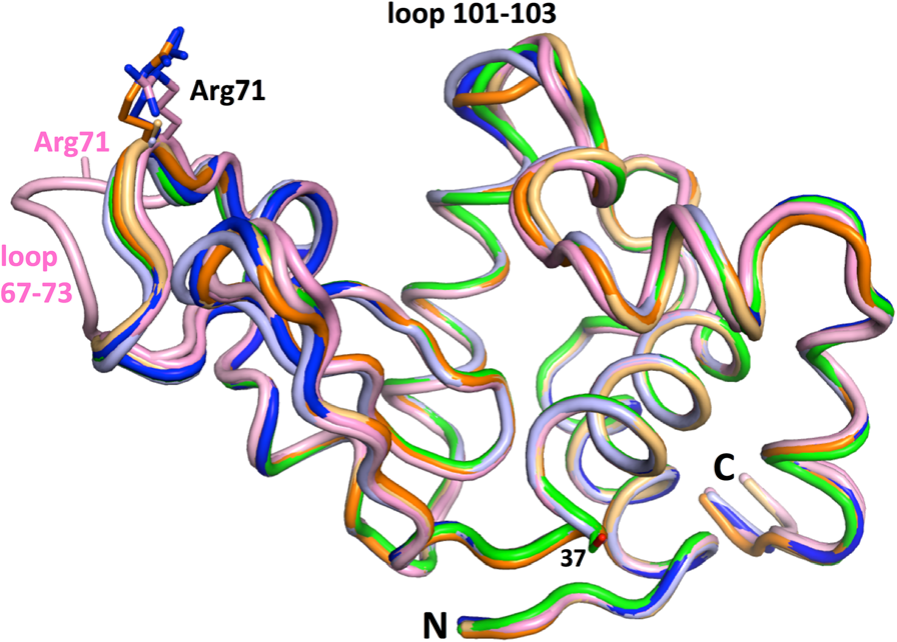
Cartoon representation of DEL-II (chains -A and -B; pink and light pink) as viewed down the catalytic cleft and superposed with DEL-I (PDB entry 5v8g, green) and DEL-III (two crystal forms: PDB entry 5v92 (orthorhombic form) coloured orange and light orange (chains -A and -B); and PDB entry 5v94 (cubic form) coloured blue and light blue (chains -A and -B)). Positions 37 and 71 are shown as sticks. The structures differ most in loop regions; loop101–103, and loop 67–73. DEL-II chain B differs most starkly from the ensemble at loop 67–73 (light pink loop and text), including Arg71, the side chain of which is disordered

The DEL-II structure comprises two C-type lysozyme molecules in the asymmetric unit (chains -A and -B). The folds of the two molecules are highly similar to each other (root mean square deviation of 0.16 Å over 99 CA positions) as well as to structures of DEL-I (PDB entry 5v8g) and DEL-III (PDB entrys 5v92 and 5v94) (see Fig. 1 for superpositions). The main difference between the folds of the chain-A and chain-B molecules are a surface loop (residues 67–73) which adopts a vastly different conformation within the chain-B molecule, relative to the chain-A molecule and the folds of molecules within structures of DEL-I and DEL-III (Fig. 1). This alternate fold is reflected by substantial differences in backbone torsion angles for this region, as compared and highlighted in Additional file 1: Table S1.

**Fig. 2:**
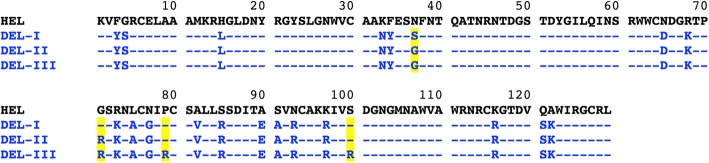
Alignment of predicted sequences of DELs -I, -II and -III (blue text) in comparison with HEL (black text), as determined by amino acid sequencing [13], DNA sequencing [12] and mass spectrometry [14]. Sequence positions identical to HEL are indicated by a dash. DEL-II is an S37G G71R double mutant of DEL-I and a R79P R100S double mutant of DEL-III (positions highlighted yellow)

The previously reported primary sequence of DEL-II, derived from amino acid and DNA sequencing as well as mass spectrometry data, predicted that it would be identical to that of DEL-I with the exception of two positions; S37G and G71R (Fig. 2, highlighted yellow) [14].

**Fig. 3:**
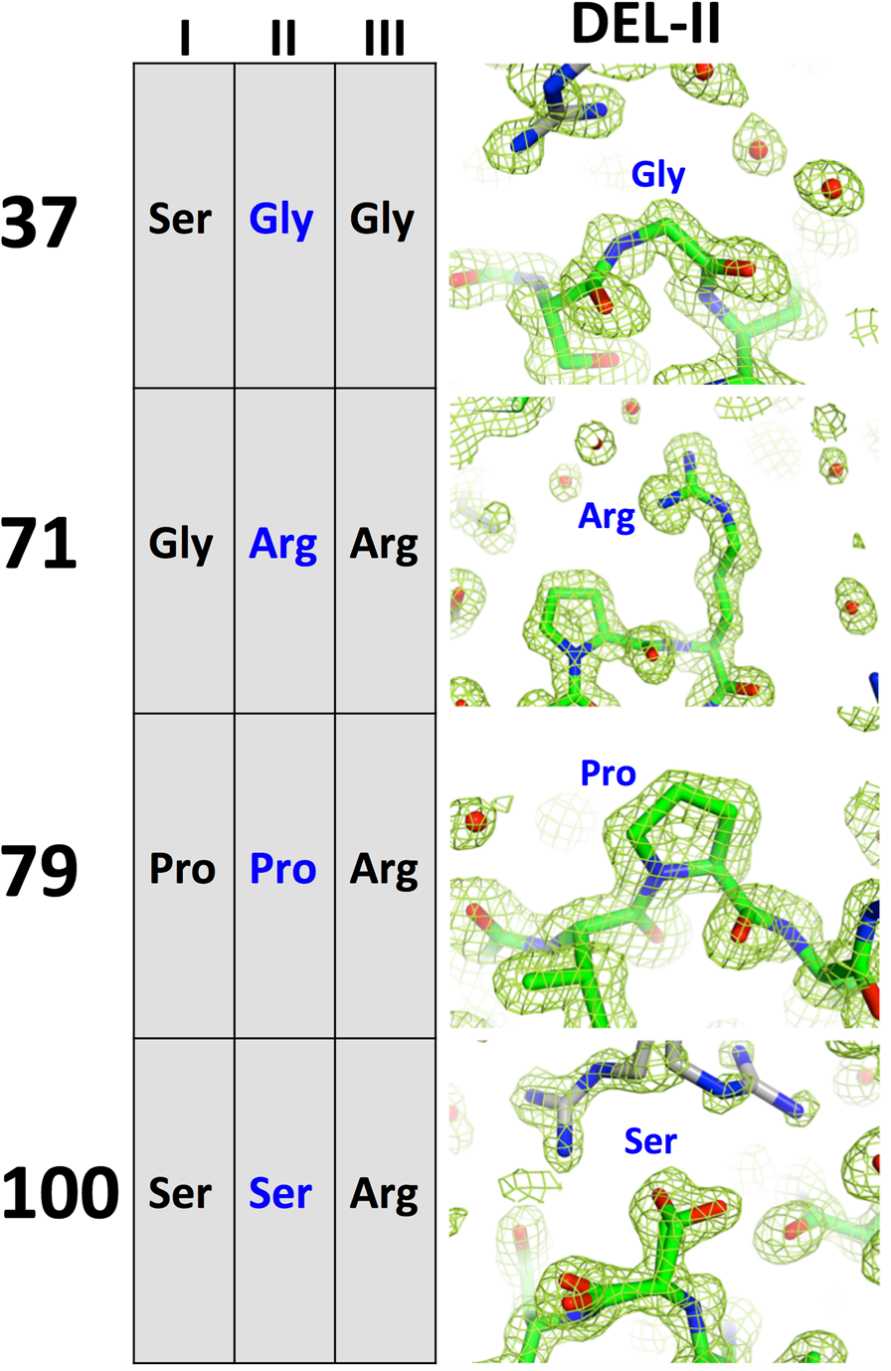
Crystallographic confirmation of DEL-II sequence. The four amino acid positions at which Pekin DEL isoforms differ (columns on the left) are compared with composite omit map electron density for the DEL-II structure (RHS, contoured at 1 standard deviation above the mean). Sticks coloured grey belong to symmetry related molecules within the crystal lattice. Sequences corresponding to the DEL-II isoform are coloured blue. At position 100, multiple conformers of Ser100 were refined (adjacent multiple conformers of Arg114 within a neighboring molecule)

Negative peaks in *f*_*obs*_*-f*_*calc*_ difference maps covering the search model (DEL-I) clearly indicated the absence of a substantive side chain at position 37 (in both chains -A and -B), consistent with glycine at this position. Conversely, strong positive *f*_*obs*_*-f*_*calc*_ electron density clearly indicated a well-ordered arginine side chain at position 71 of the A-chain (this side chain was disordered within the B-chain molecule), confirming that DEL-II is indeed an S37G G71R double mutant of DEL-I (composite omit maps shown in Fig. 3). As for DEL-I, DEL-II lacked the additional two arginine residues located at positions 79 and 100, which are observed in DEL-III (Fig. 3) [14].

**Fig. 4:**
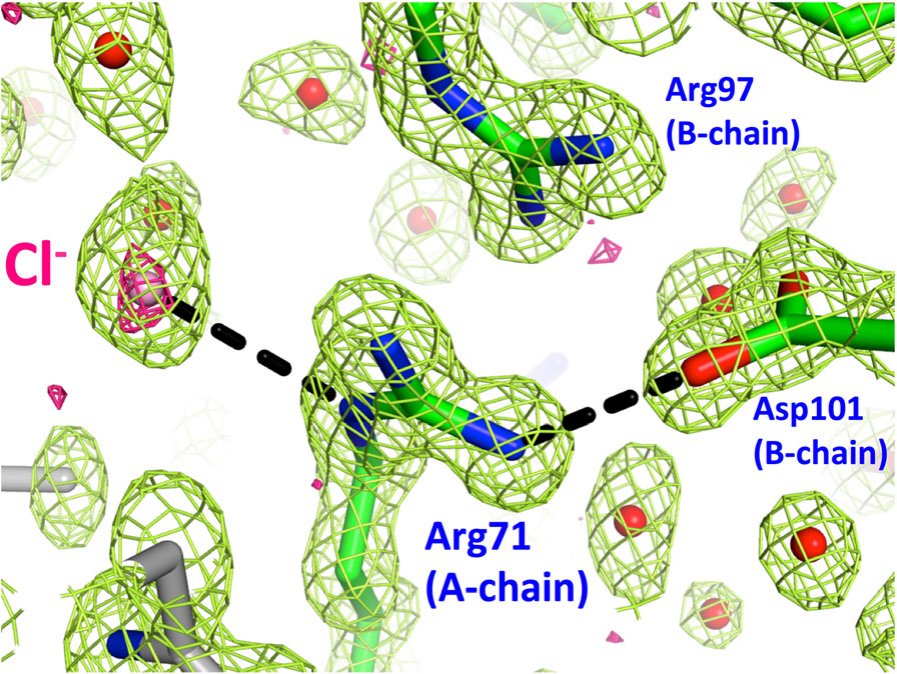
Arg71 supports different crystal packing in DEL-II relative to DEL-I. Arg71 of DEL-II projects from the A-chain molecule and interacts with the B-chain molecule by packing against Arg97 and hydrogen-bonding Asp101. It also coordinates a chloride ion (pink ball and mesh), which sits at the interface between neighboring molecules. The green mesh represents composite omit map electron density (contoured at 1 standard deviation above the mean), whilst pink mesh represents an anomalous difference map (contoured at 3 standard deviations above the mean). Sticks coloured grey belong to a symmetry related molecule within the crystal lattice

Although the quality of the electron density maps was generally extremely high (as expected at 1.15 Å resolution), there were a handful of side-chains that could not be resolved which have subsequently not been modeled (chain-A residues 47, 125 and 128; chain-B residues 45, 47, 68, 71, 122). Additionally, the electron density maps included several ‘blob’ features that we have not attempted to model (near residues Arg85/Asp87 of the A-chain, and Asn19 of the B-chain) adjacent residues clearly modeled in alternate conformations. A Mg^2+^ ion and surrounding coordinated water ligands (octahedral geometry) has been modeled adjacent residue Glu35 in both A- and B-chains. A handful of Cl^−^ ions have also been modeled, their identity confirmed by strong residual *f*_*obs*_*-f*_*calc*_ peaks on top of modeled water, generally large distances to adjacent ligands (~ 3.0–3.2 Å) and weak but discernable peaks above noise in anomalous difference maps, of magnitude similar to peaks coincident with sulfur atoms present in cysteine and methionine residues (see pink mesh in Fig. 4). Both Mg^2+^ and Cl^−^ ions were present in the crystallization conditions (~ 100 mM MgCl_2_ after dilution).

## Discussion

The structure of Pekin duck egg lysozyme isoform II (DEL-II) outlined here confirms prior sequencing and mass spectrometry data, and completes our previous structural studies of isoforms I and III [14]. Although the overall fold of DEL-II corresponds to a classical C-type lysozyme, as expected, in one of the two molecules of the asymmetric unit a surface loop of the B-chain molecule (residues 67–73) differs significantly from the A-chain molecule and from DEL-I and DEL-III, which are typical of the classic HEL fold. Centrally located within this loop is one of the four residues that together distinguish DELs -I, -II and -III, position 71. Arginine 71 in DEL-II (glycine in DEL-I) mediates interactions between the chain-A and -B molecules: the guanidinium side group of the chain-A molecule stacks against the guanidinium side chain of Arg97 of the chain-B molecule, hydrogen-bonds the side chain of Asp101 of chain-B, and it’s Nε atom coordinates an adjacent Cl^−^ ion (distance ~ 3.17 Å) held at an intermolecular interface (Fig. 4). In contrast, the side chain of Arg71 in the B-chain molecule is not observed at all in the electron density, consistent with this residue being part of a loop that packs differently within the crystal lattice compared with the A-chain molecule and with other DEL structures (Fig. 1, loop 67–73). The electron density for this B-chain loop region is weak compared to the A-chain molecule (average CA atomic B-factors of 22.7 and 12.0 Å^2^, respectively, compared with 12.8 and 11.4 Å^2^ for the full-length B- and A-chain molecules (Table 2)), with the residue at the apex of the loop (Pro70) slotting between the side chain of Asn103 of the A-chain molecule and the side chains of Asp87 and Thr89 of a symmetry-related A-chain molecule within the crystal lattice. The neighboring residue, Thr69, contributes the only direct hydrogen bonds from this loop (B-chain Thr69 O atom) to symmetry-related A-chain Arg14 atoms Nε and NH2. These DEL-II B-chain molecular contacts and mode of crystal packing are not employed in the DEL-I crystal where position 71 is replaced by a glycine residue, which, in the context of the DEL-I crystal, contributes no main-chain hydrogen bonds to crystal packing. Although the conformation of this loop is similar in DEL-II chain-A to molecules within the two crystal forms of DEL-III, the crystal contacts are, again, distinct. In both DEL-III crystal forms the side chain of Arg71 is well resolved in the A-chain molecules (whilst disordered in the B-chain molecules, which otherwise maintain the same fold), and in neither cases (for A- or B- chains) does the residue make any direct hydrogen bonds to neighboring molecules within the crystals. The diversity of fold noted for loop 67–73 (and for loop 101–103, and the C-terminus (Fig. 1)), is not uncommon within crystal structures on the surfaces of proteins, within loops connecting helices or sheets, or at polypeptide termini.

The unit cells of crystal forms of DELs -I and -II are somewhat related in that one of the unit cell dimensions is roughly equivalent (a ~ 27 Å, both space groups are *P*2_1_). We suspect that earlier attempts to obtain a DEL-II structure (scrappy interleaved crystals, poorly defined cell dimensions, multiple weak molecular replacement solutions, unconvincing maps and stalled refinement) were symptomatic of significant amounts of DEL-I contaminating the DEL-II sample and distorting lattice growth. During purification, salt-gradient mediated elution of DELs off CM resin results in DELs -I and -II partially overlapping. In terms of charge, DEL-I and DEL-II differ by just a single positive charge in the form of Arg71. Discarding DEL-II-containing fractions adjacent to those of the DEL-I peak (to three quarters peak height) presumably sufficiently removed contamination with DEL-I, allowing high-quality DEL-II crystals to be grown.

The structure of DEL-II presented is also consistent with our previous analysis of interactions between the Pekin DEL isoforms and the landmark anti-lysozyme antibodies HyHEL5 [4] and HyHEL10 [15] to which, as with DEL-I (but not DEL-III), DEL-II binds with high (K_D_ ~ 40 nM) affinity [14].

## Conclusions

Duck Egg Lysozyme (DEL) has been used as an important immunological model antigen since the 1960s. However, the exact sequences of the multiple DEL isoforms had remained ambiguous. We have recently confirmed the primary sequence of all three Pekin DEL isoforms and have reported high-resolution structures of DEL-I and -III [14]. Here we present the high-resolution structure of the remaining DEL-II isoform. Our study confirms that DEL-II corresponds to an S37G G71R variant of DEL-I, thereby completing the structural characterization of all three DEL isoforms.

## Methods

### Purification and crystallization

DEL-II was purified from DELs -I and -III, essentially as previously described [14]. Specifically, the protein was eluted from carboxy-methyl (CM) ion-exchange resin using a salt gradient (50–450 mM NaCl) in 50 mM Tris (pH 8.8) buffer. As elution peaks corresponding to DELs -I and -II (that elute sequentially) partially overlap, early fractions of the DEL-II peak (to about three quarters peak height) were discarded. Pooled fractions containing the remainder of the DEL-II peak were further chromatographed on a S200 26/60 gel-filtration column using a running buffer comprising 25 mM Tris (pH 8.0), 150 mM NaCl. Fractions containing DEL-II were pooled and concentrated to ~ 6 mg/mL. Crystallization conditions were established using a Mosquito liquid handling robot which combined 400 nL well solution with 400 nL protein solution using the commercial PACT *premier* sparse matrix screen (Molecular Dimensions). After incubation at room temperature for 1 week, plate-like crystals appeared in condition D10 (well solution comprising 200 mM MgCl_2_, 100 mM Tris (pH 8.0) and 20% (v/v) PEG6000. Crystals were removed with a nylon loop and flash frozen by plunging the loop into liquid nitrogen.

### Structure solution and refinement

X-ray diffraction data (Table 1) were recorded at the Australian Synchrotron on beamline MX2 using an Eiger × 16 M detector (Dectris). A 360 degree sweep of data was deconvoluted into 3600 images (0.1 degree each), from which reflections were indexed and integrated using iMOSFLM [16]. The space group was determined with POINTLESS [17], and scaling was performed with AIMLESS [18], both part of the CCP4 suite of software. The structure was solved by molecular replacement using PHASER [19], where 2 molecules of DEL-I (PDB entry 5v8g) were placed in the asymmetric unit (only 32% solvent). Rigid body and rounds of restrained B-factor refinement were performed with REFMAC5 [20], interspersed with inspection of models and maps enabling the correction of sequence and addition of solvent components using COOT [21]. Model refinement statistics are shown in Table 2. The high resolution of the data permitted use of anisotropic B-factors. Model validation was performed using the MOLPROBITY web server [22].

**Table 1:** Data collection and processing

Crystal	DEL-II
Diffraction source	MX2, Australian Synchrotron
Wavelength (Å)	0.9537
Spacegroup	*P*2_1_
Unit cell dimensions: a, b, c (Å); *α, β, γ, (°)*	27.34, 55.13, 69.03; *90.00, 95.95, 90.00*
Resolution range	42.99–1.15 (1.17–1.15)
Total reflections	464,884 (21,959)
Unique reflections	72,395 (3563)
Completeness (%)	100 (100)
Multiplicity	6.4 (6.2)
Average (I/σ (I))	9.0 (2.9)
Mean half set correlation, CC_(1/2)_	0.998 (0.624)
Rmeas (all I+ and I-)	0.102 (0.739)
Rpim (all I+ and I-)	0.039 (0.291)
Wilson B (Å^2^)	7.0

Data within parentheses are for the highest resolution shell

**Table 2:** Structure solution and refinement statistics

Crystal	DEL-II
R_work_/R_free_	0.155/0.186
Molecules/asu	2
Atoms protein	2074
B average protein (Å^2^)	A chain, 11.4; B chain, 12.8
Atoms water	280
B average water (Å^2^)	22.2
Atoms ions	2× Mg^2+^, 5× Cl^−^
B average ions (Å^2^)	Mg^2+^, 17.6; Cl^−^, 16.0
RMSD bond lengths (Å)	0.011
RMSD bond angles (*°*)	1.6
Ramachandran Outliers (%)	0
Ramachandran Favored (%)	99.2
PDB entry	6d9i

## Additional file

Additional file 1: **Table S1.** Backbone torsion angles for DEL-II molecules chain-A and-B, residues 60–80. Angles which differ by greater than 20 degrees between chains-A and-B are shaded yellow. (DOCX 146 kb)

## Abbreviations

DEL: Duck egg lysozyme
DEL-I: Duck egg lysozyme isoform I
DEL-II: Duck egg lysozyme isoform II
DEL-III: Duck egg lysozyme isoform III
HEL: Hen egg lysozyme
K_D_: Equilibrium binding constant
PEG: Polyethylene glycol
