# Supplementary material for: Crystal structure of duck egg lysozyme isoform II (DEL-II)
Source: BMC Struct Biol. 2018 Aug 22;18:10. doi: 10.1186/s12900-018-0090-7 (PMC6103880; doi:10.1186/s12900-018-0090-7)
Supplement: Supplementary file 1 — Table S1. Backbone torsion angles for DEL-II molecules chain-A and-B, residues 60–80. Angles which differ by greater than 20 degrees between chains-A and-B are shaded yellow. (DOCX 146 kb) [file 12900_2018_90_MOESM1_ESM.docx]

Table S1. Backbone torsion angles for DEL-II molecules chain-A and-B, residues 60-80. Angles which differ by greater than 20 degrees between chains-A and-B are shaded yellow.
